# Identification of runs of homozygosity in Western honey bees (*Apis mellifera*) using whole‐genome sequencing data

**DOI:** 10.1002/ece3.9723

**Published:** 2023-01-17

**Authors:** Annik Imogen Gmel, Matthieu Guichard, Benjamin Dainat, Geoffrey Rhys Williams, Sonia Eynard, Alain Vignal, Bertrand Servin, the Beestrong Consortium, Markus Neuditschko

**Affiliations:** ^1^ Animal GenoPhenomics, Animal Production Systems and Animal Health Agroscope Posieux Switzerland; ^2^ Swiss Bee Research Centre Agroscope Liebefeld Switzerland; ^3^ Department of Entomology and Plant Pathology Auburn University Auburn Alabama USA; ^4^ GenPhySE INRAE, INPT, INPENVT Université de Toulouse Castanet‐Tolosan France; ^5^ UMT PrADE Protection des Abeilles Dans L'Environnement Avignon France; ^6^ Domaine de Vilvert Bat 224 Jouy‐en‐Josas Cedex France

**Keywords:** *Apis mellifera carnica*, *Apis mellifera mellifera*, conservation, genetic diversity, pooled sequences, selection signatures

## Abstract

Runs of homozygosity (ROH) are continuous homozygous segments that arise through the transmission of haplotypes that are identical by descent. The length and distribution of ROH segments provide insights into the genetic diversity of populations and can be associated with selection signatures. Here, we analyzed reconstructed whole‐genome queen genotypes, from a pool‐seq data experiment including 265 Western honeybee colonies from *Apis mellifera mellifera* and *Apis mellifera carnica*. Integrating individual ROH patterns and admixture levels in a dynamic population network visualization allowed us to ascertain major differences between the two subspecies. Within *A. m. mellifera*, we identified well‐defined substructures according to the genetic origin of the queens. Despite the current applied conservation efforts, we pinpointed 79 admixed queens. Genomic inbreeding (*F*_ROH_) strongly varied within and between the identified subpopulations. Conserved *A. m. mellifera* from Switzerland had the highest mean *F*_ROH_ (3.39%), while queens originating from a conservation area in France, which were also highly admixed, showed significantly lower *F*_ROH_ (0.45%). The majority of *A. m. carnica* queens were also highly admixed, except 12 purebred queens with a mean *F*_ROH_ of 2.33%. Within the breed‐specific ROH islands, we identified 14 coding genes for *A. m. mellifera* and five for *A. m. carnica*, respectively. Local adaption of *A. m. mellifera* could be suggested by the identification of genes involved in the response to ultraviolet light (*Crh‐BP*, *Uvop*) and body size (*Hex70a*, *Hex70b*), while the *A. m. carnica* specific genes *Cpr3* and *Cpr4* are most likely associated with the lighter striping pattern, a morphological phenotype expected in this subspecies. We demonstrated that queen genotypes derived from pooled workers are useful tool to unravel the population dynamics in *A. mellifera* and provide fundamental information to conserve native honey bees.

## Graphical abstract

Past genetic diversity analyses in honey bees especially focused on drone genomes, as their haploid nature facilitated cost‐efficient whole‐genome sequencing. To substantially improve the quality of population structure analyses in honey bees we have used reconstructed queen genotypes from pooled workers to identify runs of homozygosity (ROH) in 265 Western honey bee colonies, including two subspecies namely *Apis mellifera mellifera* and *Apis mellifera carnica*. We integrated individual ROH patterns and admixture levels in a high‐resolution population network visualization, which allowed us to ascertain major differences between the two subspecies.

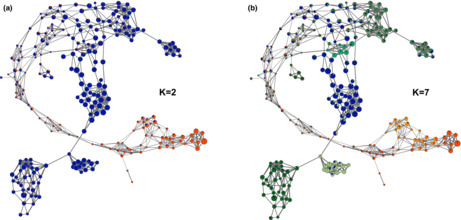

## 1 INTRODUCTION

The Western honey bee (*Apis mellifera*, hereafter honey bee*),* is a key pollinator of agricultural crops (Klein et al., 2007). To date, more than 27 subspecies have been reported globally, which can be grouped into four distinct lineages, namely M (Western and Northern Europe), C (Eastern Europe), O (Near East and Central Asia), and A (Africa; Cridland et al., 2017; Ruttner, 1988). These lineages are characterized by genetic differences leading to variable morphology, physiology, and behavior (Ruttner, 1988). Honey bees are commonly kept in hives for honey production and pollination purposes. Varying selection pressures have been applied by humans to honey bees within their native range: in Europe, several selection programs have been initiated to increase their productivity (Adam, 1983; Büchler et al., 2010; Chauzat et al., 2013; Guichard et al., 2020; Uzunov et al., 2017), while in Africa the majority of honey bees evolved without large‐scale selection (Dietemann et al., 2009).

In the beginning of the 19th century, importation of foreign honey bees among European regions began to increase, which profoundly reshaped the genetic structure of this species (Parejo et al., 2020). Historically, native honey bees of Europe mainly belong to A, M, and C evolutionary lineages. They are locally adapted to different climatic and geographical regions, resulting in several subspecies (Momeni et al., 2021; Ruttner, 1988). Nevertheless, beekeepers in Northern Europe continue to replace native honey bees (e.g., *A. m. mellifera*) with honey bees of South‐European origin (e.g., *A. m. carnica* and *A. m. ligustica*), as these subspecies are considered to be more productive, gentle and calm (Bouga et al., 2011; Guichard et al., 2021). In North America, most honey bees are hybrids of these two historically imported strains, and key selection traits in United States (US) breeding programs are productivity and resistance traits to certain pathogens (Saelao et al., 2020). In Northern Europe, the favored use of South‐European honey bees has led to multiple admixture events between subspecies and the extinction of native honey bees (Bieńkowska et al., 2021; Ruttner, 1995). Furthermore, these bees are also threatened by the widespread use of stabilized hybrid strains such as Buckfast (Adam, 1983; Bieńkowska et al., 2021).

The relocation of subspecies accompanied by admixture is a major risk factor for the loss of local adaptation and genetic diversity of honey bees (De la Rúa et al., 2009). Therefore, in Europe, several conservation programs have been initiated to maintain the genetic diversity of native honey bees, by establishing conservation areas on islands (e.g., Denmark, Scotland, and the Canary Islands) or on the mainland (e.g., France, Norway, Slovenia, and Austria), and excluding hybrids and invasive breeds mainly by their morphotype or behavior (De la Rúa et al., 2009). In Switzerland, the first conservation area of *A. m. mellifera* was established in 1977 in canton Glarus under a legal framework (Soland‐Reckeweg et al., 2009). Nowadays, an additional conservation area exists in canton Obwalden. The two conservatories encompass a total area of 830 km^2^ and ~1050 colonies (Parejo et al., 2016). To limit admixture events with other non‐native subspecies (e.g., *A. m. carnica* and Buckfast), these areas are typically located in remote alpine valleys. Besides the maintenance of the conservation areas, the breeding association of *A. m. mellifera* (mellifera.ch) established a selection program including several mating stations. These stations are also located in geographically isolated areas and consist of 10 to 20 selected drone‐producing colonies. Currently, an ancestry‐informative marker panel (microsatellites or single nucleotide polymorphisms; SNPs) is applied to determine the hybridization of conserved and selected *A. m. mellifera* queens, and queens with an admixture level greater than 10% are replaced with purebred *A. m. mellifera* (Parejo et al., 2018). However, the replacement of admixed queens is expected to lead to an increase in inbreeding that could be detrimental to the small conserved *A. m. mellifera* population. Given that the survival of honey bees is strongly dependent on their genetic diversity (Jones et al., 2004; Kryger, 1990; Mattila et al., 2012; Mattila & Seeley, 2014; Oldroyd et al., 1992), monitoring of inbreeding in small conserved populations, such as *A. m. mellifera* in Switzerland, is crucial.

Estimates of inbreeding indicate the probability that an animal receives alleles that are identical by descent from each parent. This can be estimated using genetic markers, while pedigree‐based estimations require prior knowledge of individual ancestry (Kardos et al., 2015), which in case of the honey bee is often not available. Runs of homozygosity (ROH), caused by inheritance of parental haplotypes that are identical by descent, are one of the common methods to estimate inbreeding levels without ancestry information (McQuillan et al., 2008). The length of ROH segments can be used to ascertain historical changes in population size and structure including admixture (few and short ROH segments), current inbreeding (multiple and long ROH segments), and a recent bottleneck (multiple and short ROH segments); see (Ceballos et al., 2018) for a complete review. Furthermore, it is possible to derive the genomic inbreeding coefficient (*F*_ROH_) of an animal by dividing the sum of all homozygous segments (*S*_ROH_) by the length of the analyzed genome (McQuillan et al., 2008). Numerous studies have demonstrated that overlapping ROH segments across individuals, so‐called ROH islands can be found in breed‐specific selection signatures in cattle (Purfield et al., 2012; Zhang et al., 2015), sheep (Mastrangelo et al., 2017; Purfield et al., 2017; Signer‐Hasler et al., 2019), and horses (Druml et al., 2018; Grilz‐Seger et al., 2018; Grilz‐Seger, Druml, et al., 2019; Metzger et al., 2015), as well as in cultivated plants such as avocados (Rubinstein et al., 2019), almonds (Pavan et al., 2021), and pears (Kumar et al., 2020).

To date, mostly drone genomes were used to assess the genetic diversity of honey bees, as their haploid nature facilitates cost‐efficient whole‐genome sequencing (Parejo et al., 2016). Due to the hemizygosity of drones, ROH cannot be estimated based on such data and it becomes likely to overestimate genetic relationships and subsequently inbreeding, compared to other livestock species (Wragg et al., 2016). Another disadvantage of honey bee drones is that they only explain part of the genetic diversity, as multiple paternal origins are involved in the formation of honey bee colonies (Estoup et al., 1994; Neumann et al., 1999; Tarpy et al., 2004). However, genotyping of honey bee queens for the evaluation of admixture and genomic inbreeding without harming them remains difficult (Bubnič et al., 2020; Madella et al., 2020). Therefore, a novel method for deriving queen genotypes based on pooled sequences of diploid worker bees was recently presented (Eynard et al., 2022), which could enable more genomic studies requiring diploid data in honey bees and other haplo‐diploid eusocial insects.

In this study, we investigated the utility of queen genotypes derived from pooled worker sequences to ascertain population substructures and to identify ROH segments in honey bees. Furthermore, we integrated estimates of individual admixture and *F*_ROH_ in a dynamic population network visualization to enhance the genetic monitoring of conserved *A. m. mellifera*. Finally, we screened the genomes for ROH islands to detect genes associated with geographic adaptations and human‐mediated selection within *A. m. mellifera* and *A. m. carnica*.

## 2 MATERIAL AND METHODS

### 2.1 Sampled colonies

We sampled 265 honey bee colonies from two different subspecies, namely *A. m. mellifera* (MEL) and *A. m. carnica* (CAR). Conserved MEL colonies were sampled in Switzerland (CS_CH) and France (CS_FR). The majority of the MEL colonies came from the selection program in Switzerland (SL_CH), which represents five different paternal origins (P1–P5), that is, drone‐producing colonies headed by sister queens. The sample size, geographic origin, and location of the five different paternal origins and conserved MEL colonies are summarized in Table 1. It should be noted that P1 is located in close proximity to the conservation area (CS_CH) and that P4 and P5 have a common maternal origin. The 49 sampled CAR colonies originated from Switzerland (CAR_CH, *n* = 22), Sweden (CAR_SWE, *n* = 3), Norway (CAR_NOR, *n* = 3), and the US (CAR_US, *n* = 21), while the majority of these colonies descended from open mating. For each colony, approximately 500 workers were sampled inside the hive on brood combs. Following this sample strategy, it was estimated to include all existing paternal origins among workers in the colony.

**TABLE 1:** Number of sampled colonies, geographic origin (CH = Switzerland, FR = France), legal framework and protection radius of paternal origins and conserved *Apis mellifera mellifera*

Paternal origins and conserved MEL	Number of sampled colonies	Geographic location	Canton (Ct.) and description of surroundings	Altitude (m)	Legal framework	Protection radius (distance to nearest independent apiary)
P1	17	Krauchtal, CH	Ct. Glarus, semi‐isolated valley	1400	Cantonal law: beekeepers can only breed *A. m. mellifera*	1.8 km
P2	47	Gental, CH	Ct. Bern, isolated valley	1300	Cantonal law: protection of the mating station	3 km
P3	9	Säntis, CH	Ct. Appenzell Ausserrhoden, open valley	1100	Relies on the agreement between beekeepers	2.2 km
P4	34	Schilstal, CH	Ct. St‐Gallen, semi‐isolated valley	1100	Relies on the agreement between beekeepers	3.5 km
P5	39	Rothbach, CH	Ct. Luzern, isolated valley	1300	Relies on the agreement between beekeepers	4 km
CS_CH	45	Glarus, CH	Ct. Glarus, colonies widespread across the valley (from isolated to open)	400–1000	Cantonal law: beekeepers can only breed *A. m. mellifera*	min. 4 km (distance to Canton limit)
CS_FR	25	Savoie, FR	Colonies widespread across the valley (from isolated to open)	500–1700	Relies on the agreement between beekeepers	No apiary registration

### 2.2 DNA extraction and pool sequencing

DNA extraction and pool sequencing of the sampled colonies are described in detail by Guichard et al. (2021). Briefly, approximately 500 workers per colony were shredded in a DNA extraction solution. Pair‐end sequencing was performed on an Illumina™ HiSeq 3000 or a NovaSeq™ 6000 platform. To significantly decrease computing time, the pool sequence analysis was restricted to an informative marker panel including 7,023,977 genome‐wide SNPs, as previously described by Wragg et al. (2022). Raw reads from pool sequencing of the 265 colonies were aligned to the honey bee reference genome Amel_HAV3.1, Genbank assembly accession GCA_003254395.2 (Wallberg et al., 2019). After the alignment, the resulting BAM files were converted into pileup files using the samtools mpileup utility (Li et al., 2009). Files produced by mpileup were interpreted by the PoPoolation2 utility mpileup2sync (Kofler et al., 2011) for the Sanger Fastq format, with a minimum quality of 20. Finally, sync files were converted to a depth file containing a sequencing depth value for each SNP and count files summarizing reference and alternative allele counts for each SNP.

### 2.3 Reconstruction of queen genotypes and quality control

We used the method described in Eynard et al. (2022) to reconstruct honeybee queen genotypes. In brief, this method follows a two‐step procedure using two statistical models. First, the genetic composition, in terms of proportion of the three main European honey bee subspecies (*A. m. mellifera*, *A. m. ligustica*, and *A. m. caucasica*) was estimated for each colony. For this purpose, reference allele frequencies for each of these subspecies were estimated from the data available from Wragg et al. (2022) and used as prior in the statistical model. Second, based on to estimated genetic composition (e.g., pure *A. m. mellifera*, pure *A. m. carnica* and hybrids) colonies were divided into different groups and queen genotype reconstruction was performed across colonies within such a group. On average our pool‐seq data showed the same sequencing depth (~30X), which was used to simulate the aforementioned statistical models. Therefore, we expect the same genotype errors and accuracies, previously reported by Eynard et al. (2022).

After the reconstruction of genome‐wide queen genotypes, we removed 99,555 SNPs with multiple alternative alleles and 207,904 SNPs with an excessively high and low sequencing depth. Furthermore, we excluded 771,835 homozygous SNPs to account for the very large non recombining, low polymorphic regions within the honey bee genome (Wragg et al., 2022). Finally, missing genotypes of the remaining 5,944,683 SNPs were imputed with BEAGLE 5.2 (Browning et al., 2018) to detect ROH segments along the genome, while for the population structure analyses queen genotypes were further edited for minor allelic frequency (MAF > 5%), which resulted in 1,609,447 genome‐wide SNPs.

### 2.4 Dynamic population network

To ascertain the high‐resolution population structure of honey bees, we performed a dynamic population network visualization. The different components involved in the so‐called NetView approach are described in detail by Neuditschko et al. (2012) and Steinig et al. (2016). Briefly, we computed genetic distances by subtracting pairwise relationships identical‐by‐state (IBS), as provided by PLINK v.1.9 (Chang et al., 2015), from 1 and applied the algorithm in its default setting (number of *k* nearest neighbors *k*‐NN = 10). To illustrate the genetic relatedness between neighboring honey bee queens, we associated the thickness of edges (connecting lines) with the magnitude of the genetic distance, with thicker edges corresponding to lower genetic distances. To identify highly inbred honey bee queens, we scaled the node size of each queen based on the individual *F*_ROH_. The node color denotes the sampled subpopulations and the individual level of admixture at *K* = 2 and *K* = 7 (the optimal number of clusters).

### 2.5 Admixture

Queen admixture levels and genetic distances (*F*_ST_) between the subspecies were determined using the program Admixture 1.23 (Alexander et al., 2009). We ran Admixture for 100 iterations increasing *K* from 2 to 10. Convergence between independent runs at the same *K* was monitored by comparing the resulting log‐likelihood scores (LLs) following 100 iterations, and was inferred from stabilized LLs with less than 1 LL unit of variation between runs. Cross validation error estimation for each *K* was performed to determine the optimal number of clusters. Admixture results increasing *K* from 2 to 7 were visualized with the program Distruct 1.1 (Rosenberg, 2004) and integrated in the dynamic population network, as described above.

### 2.6 Runs of homozygosity

Continuous homozygous segments were determined with an overlapping window approach implemented in PLINK v.1.9 (Chang et al., 2015) including the aforementioned 5,944,683 genome‐wide SNPs. The following settings were applied: a minimum SNP density of one SNP per 40 kb, a maximum gap length of 100 kb, and a minimum length of homozygous segment of 200 kb. The total number of ROH (*N*_ROH_), the total length of ROH segments (*S*_ROH_), and the average length of ROH (*L*_ROH_) were summarized for the two subspecies (CAR and MEL) and the respective subpopulations. The genomic‐based inbreeding coefficients (*F*_ROH_) were calculated by dividing *S*_ROH_ by the length of the autosomal genome (*L*_AUTO_), which was set to 220.76 Mb (Wallberg et al., 2019). Differences between subspecies and subpopulations were investigated using *t*‐tests (for the two subspecies) and ANOVA with post hoc Tukey's honestly significant difference (HSD) tests at a significance level of α < 0.05 as implemented in the R package multcompView (Graves et al., 2015). We also correlated *F*_ROH_ with the admixture proportions at *K* = 2 for each subspecies as implemented in the statistical computing software R (R Core Team, 2020). Furthermore, we compared *F*_ROH_ of 74 SL_CH queens with pedigree‐based inbreeding coefficients (*F*_PED_). Pedigree‐based inbreeding coefficients of the selected queens were calculated following the method described by Brascamp and Bijma (2014) based on the pedigree information of 1082 *A. m. mellifera* queens (Guichard et al., 2020) born between 1991 and 2017. The identity of the queen, of her mother and the grand‐mother (queen of the drone producing colonies) were largely known and used to establish a pedigree file, from which an inverse relationship matrix between all entries was calculated to determine *F*_PED_ (Guichard et al., 2020).

### 2.7 Homozygosity islands and gene functions

Homozygosity islands of the three different groups (CAR, SL_CH, and CS_CH) were determined based on overlapping homozygous regions present in more than 50% of the queens with <10% admixture applying the R package detectRUNS (Biscarini et al., 2019). Considering the small sample size, we used all CAR with admixture proportions <10% for the identification of breed‐specific ROH islands. Finally, we used the NCBI genome data viewer (https://www.ncbi.nlm.nih.gov/genome/gdv/), and the reference genome assembly Amel_HAv3.1 (Wallberg et al., 2019) to identify genes located in ROH islands and specified the known functions of the identified genes by conducting a literature review.

## 3 RESULTS

### 3.1 Dynamic population network

**FIGURE 1:**
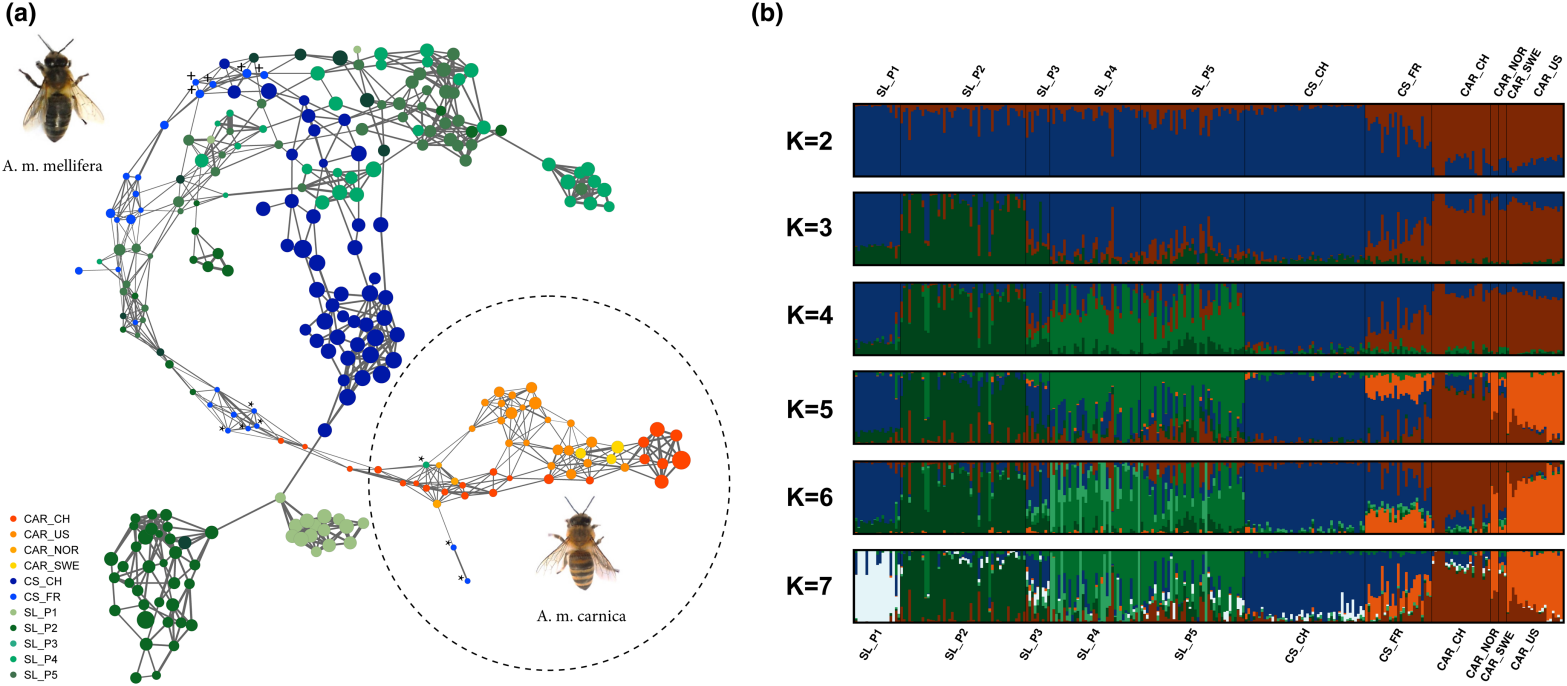
Dynamic population network and model‐based clustering of honey bees (*Apis mellifera*). (a) Dynamic population network, where each queen is illustrated by a node, with individual node size proportional to *F*_ROH_, while the node color represents the sample origin. The thickness of edges varies in the proportion of the genetic distance to visualize individual relationships between the colonies. The topology of the network clearly differentiated *Apis mellifera carnica* (CAR, dashed circle) from *Apis mellifera mellifera* (MEL) and described well‐defined substructure within MEL according to the genetic origin. MEL queens allocated in the immediate neighborhood of CAR are indicated by “*”, while CS_FR queens directly connecting with CS_CH queens are highlighted by “+”. (b) Model‐based clustering assignment of honey bees using 2–7 clusters (*K*). Queens are presented by a single vertical column divided into *K* colors. Each color represents one cluster and the length of the colored segment corresponds to the individual membership proportion in that cluster.

The dynamic population network separated CAR (Figure 1a, dashed circle) form MEL, while seven MEL queens (one SL_CH, and six CS_FR) were allocated in the immediate neighborhood of CAR (Figure 1a, indicated by “*”). The hub between CAR and MEL included CS_FR and SL_CH queens that did not cluster with their respective genetic origin. The topology of the network additionally revealed that further substructures exist within MEL queens. The most evident substructures within MEL corresponded to CS_CH queens and two selected strains (P1 and P2). It was interesting to see that five CS_FR queens (Figure 1a, top left, indicated by “+”) were directly connected with four CS_CH queens, while the remaining CS_FR queens were frequently distributed over the network. Furthermore, CS_CH queens were the nearest neighbors of five SL_CH queens originating from three different strains (P1, P4, and P5), while P1 showed the strongest genetic relationship with this cluster. Compared to P1 and P2, the three remaining strains (P3–P5) did not build a distinct population cluster. P3 queens were distributed over the network without a discernible pattern and the majority of P4 and P5 queens were highly related to each other, while especially P5 queens built two small sub‐clusters each including a P4 queen. Such a small sub‐cluster was also evident in CAR including seven highly related queens. The association of the node size with *F*_ROH_ illustrates that the majority of CS_CH and three CAR queens, included in the aforementioned sub‐cluster, showed the highest *F*_ROH_. Furthermore, it can be noted that CAR located in the neighborhood of MEL (and vice versa), as well as queens not clustering with their strains show in general lower *F*_ROH_ (Figure 1a).

### 3.2 Admixture

**FIGURE 2:**
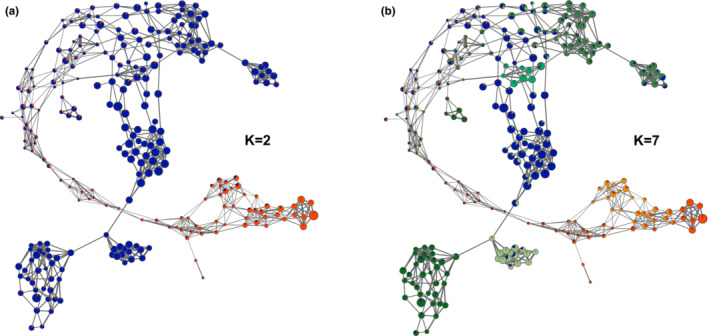
Dynamic network visualizations of honey bees (*Apis mellifera*) associated with admixture proportions. Each queen is illustrated by a node, with individual node size proportional to *F*_ROH_, while the node color represents the individual levels of admixture at *K* = 2 (a) and *K* = 7 (optimal cluster solution (b)). The thickness of edges varies in the proportion of the genetic distance to visualize individual relationship between the colonies. The topology of both networks illuminates that in general highly admixed queens also show low *F*_ROH_.

Based on the cross‐validation error estimation increasing *K* from 2 to 10, an optimal cluster solution at *K* = 7 was determined (Figure S1). The first level (*K* = 2) of model‐based clustering clearly differentiated CAR from MEL with a *F*_ST_ of 0.45 (Figure 1b). This cluster solution simultaneously highlighted that except for CS_CH and P1, all MEL subpopulations contained highly admixed queens, while CS_FR showed the highest percentage of admixed queens. At the second (*K* = 3) and third level (*K* = 4) MEL was further differentiated by allocating P2, P4, and P5 queens into two distinct clusters. At the fourth (*K* = 5) and fifth level (*K* = 6), the CAR_US queens built a distinct cluster and the common population cluster of P4 and P5 queens was further sub‐structured, without separating P4 from P5 queens. Finally, at the optimal cluster solution (*K* = 7), P1 queens were differentiated from the CS_CH cluster. Therefore, the hierarchical population clustering (increasing *K* from 2 to 7) confirmed the findings of the dynamic population network. This high agreement between the two applied population structure methods also became visible by integrating the admixture levels at *K* = 2 (Figure 2a) and *K* = 7 (Figure 2b) in the dynamic population network, which simultaneously revealed that queens not clustering with their respective geographical origin and having low *F*_ROH_ were highly admixed (Figure 2a). This observation was also reflected by an overall high negative correlation (*r* = −.75) between *F*_ROH_ and the respective admixture level of all queens (CAR and MEL) at *K* = 2. Furthermore, it can be noticed that the differentiation of the common population cluster of P4 and P5 queens at *K* = 6 was associated with one of the aforementioned subclusters (Figure 2b, center), while the hierarchical clustering failed to also detect the other one within the common cluster (Figure 2b, top right).

### 3.3 Runs of homozygosity

The number of ROH segments (*N*_ROH_), the total length of ROH (*S*_ROH_), and the *F*_ROH_ were significantly different between MEL and CAR, while the mean segment length was equal (*L*_ROH_ = 0.34 ± 0.10 Mb, Table 2). The number of ROH segments and total ROH length was nearly twice as high in MEL (*N*_ROH_ = 13.43 ± 7.21, *S*_ROH_ = 4.84 ± 2.70 Mb) as in CAR (*N*_ROH_ = 7.88 ± 5.64, *S*_ROH_ = 2.85 ± 2.27 Mb). The presence of admixture in both subspecies was reflected by the presence of queens with no ROH segments, resulting in high standard deviation (SD) values. Removing MEL queens with a CAR admixture proportion >10% at *K* = 2 (*n* = 79) increased the mean number of segments (*N*_ROH_ = 17.41 ± 4.61) and total length of segments (*S*_ROH_ = 6.30 ± 1.78) in the new subset (MEL_<10%_). Only 12 CAR queens remained after removing all samples with an admixture proportion >10% (CAR_<10%_), 10 from Switzerland (CAR_CH), and two from the US (CAR_US). The number, total length, mean length of segments, and mean genomic inbreeding coefficient all increased (*N*_ROH_ = 13.67 ± 5.16, *S*_ROH_ = 5.14 ± 2.53, *L*_ROH_ = 0.36 ± 0.06, *F*_ROH_ = 2.33 ± 1.15), but the SD increased in all parameters except for the *L*_ROH_.

**TABLE 2:** Mean values, SD, and minimum and maximum values for total number of ROH (*N*_ROH_), the total length of ROH segments (*S*_ROH_), the average length of ROH (*L*_ROH_), and genomic inbreeding coefficients (*F*_ROH_) for Apis mellifera carnica (CAR) and *Apis mellifera mellifera* (MEL)

Subspecies	Sample size	Mean	SD	Min	Max
CAR	49				
*N*_ROH_		7.88^a^	5.64	0.00	23.00
*S*_ROH_ (Mb)		2.85^a^	2.27	0.00	10.97
*L*_ROH_ (Mb)		0.34	0.10	0.00	0.51
*F*_ROH_ (%)		1.29^a^	1.03	0.00	4.97
MEL	216				
*N*_ROH_		13.43^a^	7.21	0.00	30.00
*S*_ROH_ (Mb)		4.84^a^	2.70	0.00	10.60
*L*_ROH_ (Mb)		0.34	0.10	0.00	0.66
*F*_ROH_ (%)		2.19^a^	1.22	0.00	4.80

^a^ Shows that the mean is different at a significance level of α = 0.05 based on a *t*‐test.

**FIGURE 3:**
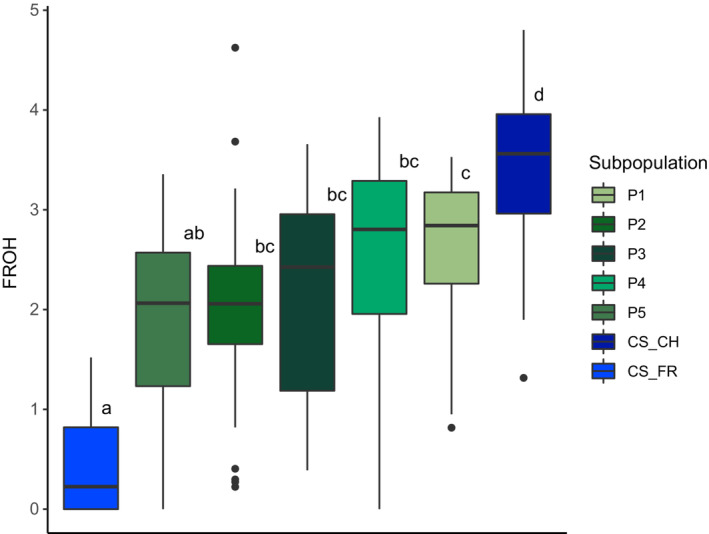
Comparison of genomic inbreeding (*F*_ROH_) between the different *A. m. mellifera* subpopulations. Boxplot of the genomic inbreeding *F*_ROH_ in percent (%) for each *A. m. mellifera* (MEL) subpopulation. The horizontal line shows the median, the box extends from the lower to the upper quartile, and the whiskers to 1.5X the interquartile range above the upper quartile or below the lower quartile. Means not sharing any letter are significantly different based on Tukey's honest different means test with a *p*‐value <.05 adjusted for multiple testing.

Summarizing the *F*_ROH_ results of all MEL queens according to the a priori defined subpopulations underscored some results from the dynamic population network (Figure 3). Pairwise Tukey's HSD comparisons between the subpopulations revealed that the CS_FR showed significantly (adjusted *p*‐value >.05) lower *F*_ROH_ (0.45 ± 0.53), while CS_CH had a significantly higher mean *F*_ROH_ (3.39 ± 0.77) compared to the other subpopulations. P1 (*F*_ROH_ = 2.63 ± 0.82) was also significantly different from P5 (*F*_ROH_ = 1.84 ± 0.93), but not from P2 (*F*_ROH_ = 1.98 ± 0.87), P3 (*F*_ROH_ = 1.84 ± 0.93), and P4 (*F*_ROH_ = 2.39 ± 1.18). P2, P3, and P4 were neither significantly different from each other nor from P5. There were no significant differences in ROH values between the CAR subpopulations due to the small sample size.

The *F*_PED_ values, of 74 SL_CH queens including all strains except P4, ranged from 0.00 to 5.18%, with a mean of 1.65% ± 1.41, whereas *F*_ROH_ ranged from 0.00 to 4.62%, with a mean of 2.08% ± 0.95. The correlation between *F*_PED_ and *F*_ROH_ was slightly negative (*r* = −.22).

### 3.4 Homozygosity islands

After exclusion of all MEL queens with an admixture level greater than 10% at *K* = 2, we considered two subpopulations, SL_CH_<10%_ (*n* = 94), representing the selection lines, and CS_CH_<10%_ (*n* = 43) from the Swiss conservation area. We identified 15 SL_CH_<10%_‐specific homozygosity islands distributed over nine chromosomes (Table S1). Considering only the CS_CH_<10%_ queens there were 19 islands, with more on chromosomes 9 and 11, but fewer on chromosomes 6 and 10 than for SL_CH_<10%_ queens, and one on chromosome 15 (Table S2). Twelve islands were overlapping, on chromosomes 1, 2, 3, 5, 6, 8, 9, and 11. Interestingly, there were islands on chromosomes 8, 9, 10, and 11 appearing in SL_CH_<10%_ queens that were not present in CS_CH_<10%_ queens.

Chromosomes 4, 7, 12, 13, 14, and 16 did not bear any homozygosity islands for either MEL subpopulation. Five homozygosity islands were located near the starting end of the chromosomes. The largest homozygosity island was on chromosome 2 for CS_CH and covered 943 kb (common with SL_CH_<10%_ over two smaller regions of 16 and 36 kb, respectively). The shortest was for MEL_<10%_ on chromosome 3 and spanned 15 kb. There were substantially more uncharacterized genes than annotated genes within the ROH islands (i.e., 264 uncharacterized loci and 12 annotated genes in MEL_<10%_, respectively, 447 and 10 for CS_CH). Table 3 summarizes the annotated genes embedded in the ROH islands.

**TABLE 3:** Location and length of homozygosity islands (runs of homozygosity shared by >50% of individuals) for *Apis mellifera mellifera* and *Apis mellifera carnica* containing characterized genes

Chr.	Start	End	Length (kb)	Subpopulation	Genes
2	2,030,227	2,065,958	35.73	CS_CH_<10%_ & SL_CH_<10%_	***Tert***
3	309,688	692,594	382.91	CS_CH_<10%_	***Ndufs1***
5	3723	446,000	442.28	CS_CH_<10%_	***Phrf1***
5	483,461	656,850	173.39	CS_CH_<10%_	***Chmp1***
8	2,038,809	2,354,163	315.35	SL_CH_<10%_	***Rpl35, Ctl5,*** ***Crh‐BP**, **ATP5G2***
8	2,355,518	2,416,454	60.94	SL_CH_<10%_	***Tmem98**, **Twi***
8	11,659,873	11,857,385	197.51	CS_CH_<10%_ & SL_CH_<10%_	***Hex70a, Hex70b***
8	11,857,385	11,857,837	0.45	CS_CH_<10%_	***Hex70b***
9	842,007	1,263,372	421.37	CS_CH_<10%_	***Grp**, **Rep***
9	1,812,584	1,983,997	171.41	CS_CH_<10%_ & SL_CH_<10%_	***WRNexo***
10	189,237	414,181	224.94	SL_CH_<10%_	***Uvop***
11	6,294,671	6,757,043	462.37	CAR_<10%_	***Cpr1, Cpr2, Cpr3, Cpr4, Rga***
15	2,635,510	2,869,345	233.84	CS_CH_<10%_	***Snf***

*Note*: SL_CH_<10%_ are all A. m. mellifera queens with less than 10% admixture proportions from the selection lines, CS‐CH_<10%_ are MEL queens from the Swiss conservation area with less than 10% admixture proportions, CAR_<10%_ are the A. m. carnica queens with less than 10% admixture proportions.

*Tert:* Telomerase Reverse Transcriptase*, Ndufs1:* NADH dehydrogenase (ubiquinone) Fe‐S protein 1, 75 kDa (NADH‐coenzyme Q reductase), *Phrf1*: PHD and RING finger domain‐containing protein 1, *Chmp1:* chromatine modifying protein 1, *RpL35:* ribosomal protein L35, *Ctl5:* C‐type lectin 5, *Crh‐BP:* corticotropin‐releasing hormone binding protein, *ATP5G2:* ATP synthase H+ transporting mitochondrial F_0_ complex, subunit C2 (subunit 9), *Tmem98*: transmembrane protein 98, *Twi:* Twist, *Hex70A*: Hexamerin 70A, *Hex70B*: Hexamerin 70B, *Grp*: glycine‐rich cuticle protein, *Rep:* Rab escort protein, *Wrnexo*: WRN exonuclease, *Uvop*: ultraviolet‐sensitive opsin, *Cpr*: cuticular protein, *Rga:* regulator of gene activity protein, *Snf*: U1 small nuclear ribonucleoprotein A.

The genes *Ndufs1*, *Phrf1*, *Chmp1*, *Grp*, *Rep*, and *Snf* were in homozygosity islands specific to CS_CH queens. The genes *RpL35*, *Ctl5*, *Crh‐BP*, *ATP5G2*, *Tmem98*, *Twi*, and *Uvop* were in a homozygosity island specific to SL_CH queens.

For the 12 purebred CAR queens (CAR_<10%_) we identified 11 ROH islands, all on chromosome 11, roughly spanning from 4,235,653 to 7,082,258 bp (Table S3). The shortest island was 25 kb and the longest island was 499 kb. The second longest, with 462 kb, contained five genes coding for cuticular proteins: *Cpr1*, *Cpr2*, *Cpr3*, and *Cpr4*, as well as a regulator of gene activity protein (*Rga*, Table 3). Similarly, to MEL, the homozygosity islands contained mainly uncharacterized loci (105) compared to the five annotated genes.

## 4 DISCUSSION

We demonstrated that queen genotypes derived from pooled honey bee workers can be successfully applied to ascertain high‐resolution population structures, including the computation of *F*_ROH_ and the detection of breed‐ and subpopulation‐specific ROH islands. However, it should be noticed, that the applied queen reconstruction procedure assumes that queens are mated to drones of similar ancestry. Our pool‐seq data, where the majority of colonies were derived from breeders and conservatories fulfilled this prerequisite, while for other data settings (e.g., artificial insemination), the applicability of the queen reconstruction procedures needs to be further investigated. The applied population structure analyses clearly differentiated MEL from CAR (*F*_ST_ = 0.45), despite the occurrence of highly admixed MEL and CAR queens and simultaneously highlighted the challenges to conserve native honey bees due to lack of control over mating. Therefore, the dynamic population network illustrated that a successful honey bee conservation program requires an appropriate management tool including a legal framework, a suitable geographical isolated location, and ancestry informative marker testing, like the conservation strategy of MEL in the Canton Glarus, the only area without highly admixed colonies (CS_CH and P1). However, in our view the strong gene flow between the two subpopulations can have a negative impact on the in situ conservation as selected P1 queens might introduce foreign genetic variants to the CS_CH gene pool.

Compared to CS_CH, the origin of CS_FR queens was only sporadically assessed in the past based on wing vein measurements, which simultaneously explains the highly observed diversity of the queens, whereas six queens showed a high genetic relatedness with CAR. The population structure of CS_FR and the genetic origin of some SL_CH indicate that current applied conservation strategies including the geographical locations are not suitable for in situ conservation. Ex situ conservation by means of artificial insemination (Cobey et al., 2013), could be a more efficient alternative to maintain the gene pool of native honey bees.

In spite of the fact that SL_CH are carefully selected to contribute to the local genetic diversity, MEL showed significantly higher *F*_ROH_ than CAR. However, this comparison must be moderated, as the majority of CAR queens originated from open mating not following a clear selection. This characteristic of CAR samples was also evident in the dynamic population network, which illuminated the high admixture levels and low *F*_ROH_ of CAR queens, whereas it was also possible to identify some purebred CAR, which showed similar *F*_ROH_ compared to MEL. Hence, for a comprehensive comparison between MEL and CAR population structure, further investigations are needed, especially involving more selected CAR queens.

The ROH results, according to the observed population structure (Figure 1a), confirmed the direct inverse relationship (*r* = −.75) between admixture and ROH length in honey bees: there were fewer and shorter ROHs in queens with higher admixture proportions, concurrent to previous findings on the individual level in other livestock populations such as cattle (Purfield et al., 2012) and goats (Bertolini et al., 2018). The population admixture also had an effect on the relationship between *F*_ROH_ and *F*_PED_, with a slightly negative correlation (*r* = −.22) indicating poor agreement between the two methods, compared to commonly observed values in livestock, such as goats (*r* = .50; Burren et al., 2016) and sheep (0.18 < *r* < .70; Purfield et al., 2017). In absence of instrumental insemination, the paternal origin of honey bees is not precisely known, as honey bee queens naturally mate in flight with 10 to 20 drones (polyandrous mating system; Estoup et al., 1994; Neumann et al., 1999; Tarpy et al., 2004). Hence, the paternal origin must be estimated by restricting paternal origins to the drone‐producing colonies located at the mating station. However, the proportion of foreign drones contributing to the mating remains unknown. Therefore, *F*_PED_ of queens from insufficiently isolated mating stations (with higher admixture proportions) is overestimated, while a low pedigree completeness results in lower *F*_PED_ compared to *F*_ROH_. To improve the pedigree quality of honeybees, we suggest confirming the parental origin with a marker‐based parentage analysis or by performing artificial insemination.

Within MEL‐specific homozygosity islands, we identified two genes that are directly associated with the current applied selection traits, including increased productivity and swarming drive (Bouga et al., 2011; Guichard et al., 2021). Based on highly selected *A. m. ligustica* strains, it has already been demonstrated that *RpL35*, identified in the SL_CH_<10%_‐specific island, controls royal jelly production and larval growth (Ararso et al., 2018). Furthermore, the differential expression of *Ndufs1*, found in a CS_CH_<10%_‐specific ROH island, may also increase foraging behavior (Guo et al., 2019), and consequently, productivity.

The gene *Crh‐BP* embedded in a ROH island for SL_CH_<10%_, is involved in the resistance to ultraviolet (UV) exposure, and therefore suggests adaptive mechanisms due to the ancestral geographical origin of the subspecies. The gene *Crh‐BP* was shown to be upregulated in honey bees in response to UV exposure and heat stress (Even et al., 2012). Therefore, homozygosity in this gene could indicate local adaptation to lower sun exposure and temperatures of Northern Europe by potential downregulation of this gene. The homozygous state of the *Uvop* gene in SL_CH_<10%_ is associated with retinal development and the circadian rhythm (Lichtenstein et al., 2018), which may enable SL_CH_<10%_ to deal with seasonally more variable sun exposure. Diurnal mammal species also produce different quantities of UV‐sensitive pigments depending on their ecological niche (Emerling et al., 1819). Furthermore, it has recently been demonstrated that genes involved in the response to UV exposure are associated with the local adaptation of horse breeds (Grilz‐Seger, Neuditschko, et al., 2019).

Another indication of signatures of selection related to the geographical distribution of MEL can be found in the homozygosity of the genes *Hex70a* and *Hex70b*, encoding for two hexamerin proteins of the same name. Similar to vitellogenin, hexamerins bind to juvenile hormone (JH) and are storage proteins in the larval fat body, providing amino acids for the development into the adult stage (Martins et al., 2010; Telfer & Kunkel, 1991). Hexamerins also appear to be involved in ovary and testes development, and spermatogenesis in drones (Martins et al., 2011). More storage proteins in the larval fat body imply both larger and more long‐lived bees, essential for colony survival during the winter. Although the quantity of storage proteins mostly depends on the pollen supply and quality (Basualdo et al., 2013; Frias et al., 2016), differences between queen strains have been observed under comparable feeding conditions, suggesting a genetic component (DeGrandi‐Hoffman et al., 2021). We stipulate here that the ROH islands containing *Hex70a* and *Hex70b* could be linked to protein conversion efficiency, body size, and longevity of queens, allowing MEL to survive a longer winter period. This would be consistent with Bergman's rule, predicting that larger animals are better adapted to colder conditions (see Chole et al. (2019) for a review on bee size). The evolutionary emergence of longer‐lived workers accumulating vitellogenin, another JH‐binding protein, in MEL and CAR subspecies compared to the subtropical *A. m. scutellata* subspecies would suggest a similar adaptation in the quantity of accumulated hexamerin proteins in MEL (northern origin) compared to CAR (southern origin; Seehuus et al., 2006). Ruttner described both MEL and CAR as “large” (Ruttner, 1988), therefore objective studies measuring multiple workers from diverse *Apis mellifera* subspecies are necessary to confirm this hypothesis.

Several characterized genes in a homozygous state for MEL shared functions associated with stress response (*ATP5G2* (Watts et al., 2018), *Crh‐BP* (Even et al., 2012), *Hex70b* (Aronstein et al., 2010)), and immunity (*Ctl5* (Lin et al., 2020)). Two genes, *Wrnexo* and *Tert*, are involved in DNA structure and integrity, and therefore are thought to be associated with longevity (Hornstein, 2008; Robertson & Gordon, 2006; Rossi et al., 2010). However, at the current stage of research, it is not clear whether the homozygosity state of these genes has a positive or negative effect on the aforementioned functions. Therefore, fine‐tuned gene expression studies are required to assess the direction of selection within the MEL subspecies.

We also identified genes related to the exterior phenotype used to distinguish the two subspecies. The gene *Chmp1* identified in a CS_CH_<10%_ ROH island is known to influence the veining pattern in *Drosophila* (Valentine et al., 2014), which might explain the morphological differences in vein patterns used to classify individuals into subspecies (Bouga et al., 2011; Ruttner, 1988). Several cuticular protein‐coding genes (*Cpr3* and *Cpr4* in particular), present in the private ROH island of CAR_<10%_ may be involved in the CAR‐specific morphotype of broader hairy stripes (Figure 1a), as they affect the thickness and coloring of the exoskeleton (Costa et al., 2016; Soares et al., 2013). We found another gene related to the cuticle (*Grp*, glycine‐rich cuticle protein) in a CS_CH_<10%_‐specific island, although its functions have not been formally described in the literature as far as we could discern. The *Twi* gene is involved in establishment of both the anterior–posterior and dorsal‐ventral axes during embryogenesis, including the segmentation (i.e., stripes) of the abdomen (Wilson et al., 2014). Whether species‐specific homozygosity in this gene could affect broadness of stripes was not specified. The identified genes discussed here are only a fraction of the loci found in the homozygosity islands for either MEL or CAR. The poor annotation of the current reference genome does not allow for a more thorough interpretation of our results.

In summary, we have described a number of novel aspects to investigate the genetic diversity of honey bees that are of potential interest. First, the application of queen genotypes derived from pooled honey bee workers to ascertain fine‐scale population structures. Second, the identification of ROH segments to compute genomic inbreeding of honey bee queens. Finally, the identification of genes associated with geographic adaptation and human‐mediated selection by means of ROH islands. Therefore, we believe that ROH derived from whole‐genome sequencing data will be of invaluable benefit to investigate complex population structures in honey bees and other insects.

## AUTHOR CONTRIBUTIONS

**Annik Imogen Gmel:** Formal analysis (equal); visualization (equal); writing – original draft (equal). **Matthieu Guichard:** Data curation (equal); formal analysis (equal); investigation (equal); visualization (equal); writing – original draft (equal). **Benjamin Dainat:** Conceptualization (equal); data curation (equal); supervision (equal); writing – review and editing (equal). **Geoffrey Rhys Williams:** Data curation (equal); writing – review and editing (equal). **Sonia Eynard:** Data curation (equal); methodology (equal); writing – review and editing (equal). **Alain Vignal:** Data curation (equal); methodology (equal); writing – review and editing (equal). **Bertrand Servin:** Data curation (equal); methodology (equal); writing – review and editing (equal). **the Beestrong Consortium:** Data curation (equal); writing – review and editing (equal). **Markus Neuditschko:** Conceptualization (lead); data curation (equal); formal analysis (lead); funding acquisition (equal); supervision (equal); writing – original draft (equal); writing – review and editing (equal).

## FUNDING INFORMATION

Financial support for this study was provided by Bundesamt für Landwirtschaft BLW (Swiss Federal Office for Agriculture FOAG) Grant No. 627000708, by Labogena and FranceAgriMer (Programme d'Investissements d'Avenir), the Alabama Agricultural Experiment Station, and the USDA NIFA Multi‐state Hatch Project NC1173.

## Supporting information


Figure S1

Table S1

Table S2

Table S3

## Data Availability

Swiss, Swedish, Norwegian, and American bee sequence data will be deposited at the European Nucleotide Archive (ENA: http://www.ebi.ac.uk/ena), while French bee sequence data remain the property of the Beestrong Consortium. However, data are available from the authors upon reasonable request.
