# Supplementary figures and images for: Identification of runs of homozygosity in Western honey bees (Apis mellifera) using whole‐genome sequencing data
Source: Ecol Evol. 2023 Jan 17;13(1):e9723. doi: 10.1002/ece3.9723 (PMC9843643; doi:10.1002/ece3.9723)

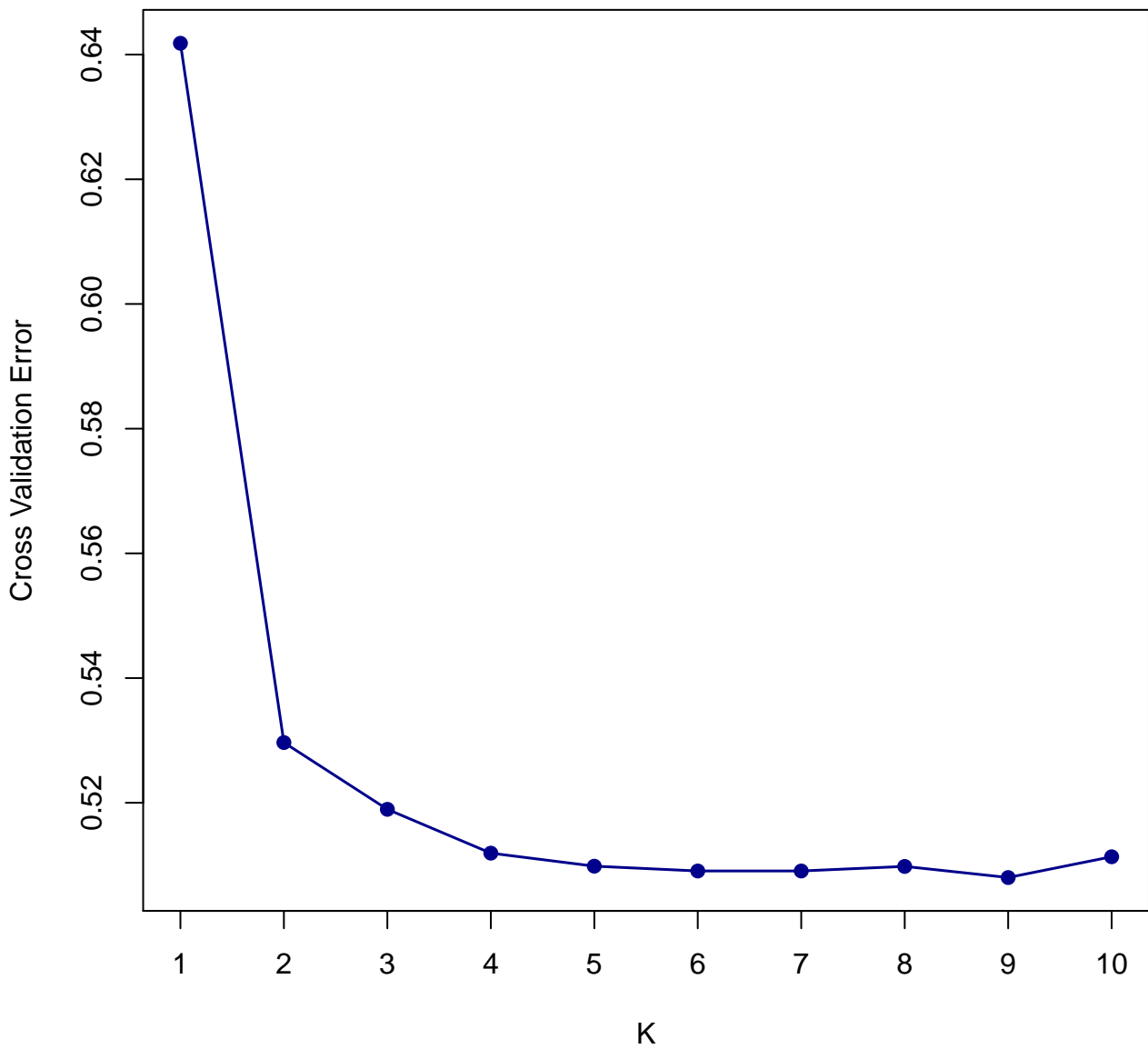

Supplement: Supplementary file 1 — Figure S1 [file ECE3-13-e9723-s002.pdf]
