# Supplementary material for: Identification of runs of homozygosity in Western honey bees (Apis mellifera) using whole‐genome sequencing data
Source: Ecol Evol. 2023 Jan 17;13(1):e9723. doi: 10.1002/ece3.9723 (PMC9843643; doi:10.1002/ece3.9723)
Supplement: Supplementary file 2 — Table S1 [file ECE3-13-e9723-s004.docx]

Table S1 : List of annotated and uncharacterised loci within homozygosity islands of *Apis mellifera mellifera* from the selection lines (P1-P5) with admixture proportions < 10% (n=94)

| **Chr.** | **Begin (bp)** | **End (bp)** | **Length (Kb)** | **N annot. genes** | **N. unchar. loci** | **Characterised genes and uncharacterised loci** |
| --- | --- | --- | --- | --- | --- | --- |
| 1 | 11,536,425 | 11,764,891 | 228.47 | 0 | 36 | *LOC100576131, LOC412112, LOC100579052, LOC724620, LOC551107, LOC726944, LOC411997, LOC552371, LOC726918, LOC412748, LOC409079, LOC727390, LOC412495, LOC550977, LOC727396, LOC102654495, LOC100578820, LOC412721, LOC724633, LOC724678, LOC552473, LOC726264, LOC552497, LOC551976, LOC551785, LOC412636, LOC100578966, LOC100578479, LOC726667, LOC409351, LOC726694, LOC100577197, LOC726722, LOC409860, Mir3770, LOC113219403* |
| 2 | 1,921,718 | 1,937,794 | 16.08 | 0 | 2 | *LOC100576649, LOC409111* |
| 2 | 2,030,227 | 2,065,958 | 35.73 | 1 | 1 | *LOC100576862,* ***Tert*** |
| 3 | 70,566 | 294,129 | 223.56 | 0 | 10 | *LOC409422, LOC107965799, LOC100576748, LOC102656479, LOC724483, LOC412045, LOC724402, LOC724363, LOC724270, LOC551485* |
| 3 | 294,878 | 309,688 | 14.81 | 0 | 4 | *LOC551485, Mir6047a, Mir6047b, LOC726869* |
| 5 | 446,000 | 483,461 | 37.46 | 0 | 4 | *LOC726916, LOC100576349, LOC551550, LOC551520* |
| 6 | 137,912 | 289,489 | 151.58 | 0 | 1 | *LOC100578680* |
| 8 | 2,038,809 | 2,354,163 | 315.35 | 5 | 31 | *LOC102654371, LOC100577229, LOC100577302,* ***RpL35, CTL5,*** *LOC409195, LOC409196, LOC411771, LOC409369, LOC409370, LOC409371,* ***crh-BP****, LOC724393, LOC724437, LOC724533, LOC410085, LOC411881, LOC409250, LOC724519, LOC551603, LOC551301,* ***ATP5G2****, LOC411658, LOC552596, LOC100576968, LOC409150, LOC727647, LOC727649, LOC409165, LOC409166, LOC726297, LOC411698, LOC107964792, LOC409325, LOC409836, LOC102654020* |
| 8 | 2,355,518 | 2,416,454 | 60.94 | 2 | 10 | *LOC409836, LOC102654020, LOC113218966, LOC413252,* ***Tmem98****, LOC413253,* ***Twi****, LOC100576319, LOC107963998, LOC100577452, LOC100576382, LOC726359* |
| 8 | 11,659,873 | 11,857,385 | 197.51 | 2 | 29 | *LOC551263, LOC409912, LOC551308, LOC551696, LOC726007, LOC551754, LOC412192, LOC550698, LOC100576975, LOC727634, LOC412069, LOC552284, LOC412398, LOC100578892, LOC725039, LOC411989, LOC102656541, LOC551376, LOC100578450, LOC107964839, LOC724870, LOC102656792, LOC409637, LOC726778, LOC551888, LOC412504, LOC726811, LOC726822, LOC409353,* ***Hex70a, HEX70b*** |
| 9 | 1,459,383 | 1,812,243 | 352.86 | 0 | 41 | *Mir6006, LOC107965007, LOC412647, LOC727153, LOC727157, LOC726740, LOC552467, LOC409741, LOC552273, LOC102654498, LOC552291, LOC551554, LOC551576, LOC551907, LOC107965832, LOC551936, LOC100576439, LOC102656685, LOC412510, LOC100576436, LOC411960, LOC550780, LOC551445, LOC725807, LOC102656492, LOC550692, LOC551097, LOC100576348, LOC727599, LOC411653, LOC409144, LOC725563, LOC413984, LOC113218977, LOC100578763, LOC725558, LOC551433, LOC412446, LOC102656849, LOC412787, LOC113218985* |
| 9 | 1,812,480 | 2,064,783 | 252.30 | 1 | 9 | *LOC412787, LOC102656780, LOC726239,* ***WRNexo****, LOC413692, LOC550940, LOC727278, LOC412688, LOC726367, LOC100576170* |
| 10 | 189,237 | 414,181 | 224.94 | 1 | 30 | *LOC552681, LOC102656733, LOC552669, LOC412281, LOC100576438, LOC551414, LOC413989, LOC551353, LOC727262, LOC550715, LOC100576270, LOC411655, LOC411654, LOC410060,* ***Uvop****, LOC102655648, LOC102655612, LOC724822, LOC551231, LOC725224, LOC100577607, LOC551999, LOC551953, TRNAM-CAU, LOC727288, LOC100577690, LOC551901, LOC412053, LOC727275, LOC727269, LOC412051* |
| 11 | 4,337,589 | 4,449,530 | 111.94 | 0 | 13 | *LOC725069, LOC552002, LOC411811, LOC727122, LOC727290, LOC412423, LOC725316, LOC113219102, LOC100578943, LOC409438, LOC727299, LOC107965795, LOC113219105* |
| 11 | 14,818,859 | 15,025,079 | 206.22 | 0 | 43 | *LOC410154, LOC551424, LOC724842, LOC724877, LOC409595, LOC409877, LOC552769, LOC100577344, LOC412219, LOC727234, LOC409393, LOC552744, LOC727222, LOC551313, LOC552733, LOC411505, LOC409491, LOC552714, LOC413822, LOC552700, LOC551276, LOC410108, LOC727221, LOC412046, LOC409329, LOC100576647, LOC551964, LOC107965281, LOC725781, LOC409241, LOC411861, LOC411862, LOC411863, LOC409636, LOC409634, LOC102653856, LOC100576717, LOC551467, LOC107965192, LOC409838, LOC409839, LOC413259, LOC100578596* |
