# Supplementary material for: Identification of runs of homozygosity in Western honey bees (Apis mellifera) using whole‐genome sequencing data
Source: Ecol Evol. 2023 Jan 17;13(1):e9723. doi: 10.1002/ece3.9723 (PMC9843643; doi:10.1002/ece3.9723)
Supplement: Supplementary file 3 — Table S2 [file ECE3-13-e9723-s003.docx]

Table S2 : List of annotated and uncharacterised loci within homozygosity islands of Apis mellifera mellifera from the conservation area in Switzerland with <10% admixture (n=43)

| **Chr.** | **Begin (bp)** | **End (bp)** | **Length (Kb)** | **N annot. genes** | **N. unchar. loci** | **Characterised genes and uncharacterised loci** |
| --- | --- | --- | --- | --- | --- | --- |
| 1 | 11,536,425 | 11,764,891 | 228.47 | 0 | 36 | *LOC100576131, LOC412112, LOC100579052, LOC724620, LOC551107, LOC726944, LOC411997, LOC552371, LOC726918, LOC412748, LOC409079, LOC727390, LOC412495, LOC550977, LOC727396, LOC102654495, LOC100578820, LOC412721, LOC724633, LOC724678, LOC552473, LOC726264, LOC552497, LOC551976, LOC551785, LOC412636, LOC100578966, LOC100578479, LOC726667, LOC409351, LOC726694, LOC100577197, LOC726722, LOC409860, Mir3770, LOC113219403* |
| 2 | 1,130,935 | 1,345,282 | 214.35 | 0 | 5 | *LOC100576732, LOC724673, LOC102655218, LOC726292, TRNAA-UGC* |
| 2 | 1,571,346 | 2,514,393 | 943.05 | 1 | 42 | *LOC102654800, Mir3757, LOC727515, LOC727168, LOC727350, LOC113219428, LOC727358, LOC726925, LOC411359, LOC552129, LOC727574, LOC113219424, LOC100578586, LOC113218599, LOC107965774, LOC409793, LOC409489, LOC726110, LOC100576649, LOC409111, LOC100576896, LOC100576862,* ***Tert****, LOC100576829, LOC725528, LOC412594, LOC725850, LOC727119, Mir3762, LOC102655454, LOC552202, LOC100578587, LOC412646, LOC726171, LOC102654530, LOC102654576, LOC412465, LOC102654729, LOC100217360, LOC102654685, LOC100579011, LOC100578724, LOC102655559* |
| 3 | 45,010 | 692,594 | 647.58 | 1 | 49 | *LOC409422, LOC102654728, LOC107965799, LOC100576748, LOC102656479, LOC724483, LOC412045, LOC724402, LOC724363, LOC724270, LOC551485, Mir6047a, Mir6047b, LOC726869, LOC413233, LOC727354, LOC551755, LOC113218664, LOC410161, LOC413372, LOC102655050, LOC552667, LOC412506,* ***Ndufs1****, LOC552649, LOC409663, LOC100576775, LOC552241, LOC552175, LOC100576120, LOC727415, LOC409242, LOC551723, LOC411536, LOC100576296, LOC551966, LOC412619, LOC100576506, LOC552280, LOC113218653, LOC113218654, LOC113218655, LOC409556, LOC551442, LOC412432, LOC102654682, LOC100576930, LOC552200, LOC409492, LOC550978* |
| 5 | 3,723 | 656,850 | 653.13 | 2 | 65 | *LOC725909, LOC551311, LOC412401, LOC551339, LOC412644, LOC724370, LOC724529, LOC412104, LOC551185, LOC552161, LOC409589, LOC552182, LOC100577579, LOC552227, LOC726900, LOC409283, LOC551466, LOC727257, LOC552720, LOC412676, LOC412268, LOC413815,* ***PHRF1****, LOC551867, LOC551519, LOC100577094, LOC725529, LOC724831, LOC102653815, LOC412245, LOC409796, LOC413757, LOC113218560, Mir3759, LOC107965828, LOC725409, LOC100578534, LOC725337, LOC725303, LOC725267, LOC414011, LOC725098, LOC113218814, LOC552402, LOC726916, LOC100576349, LOC551550, LOC551520, LOC100576378, LOC726058, LOC726501, LOC552572, LOC409581, LOC552566, LOC100576886, LOC113218767, LOC727141, LOC100576155,* ***Chmp1****, LOC727432, LOC413809, LOC727639, LOC551782, LOC113218783, LOC727022, LOC551464, LOC727035* |
| 6 | 1,031 | 524,080 | 523.05 | 0 | 4 | *LOC113218872, LOC113218833, LOC100578680, LOC102653843* |
| 8 | 11,635,451 | 11,857,837 | 222.39 | 2 | 32 | *LOC551131, LOC725023, LOC409529, LOC551263, LOC409912, LOC551308, LOC551696, LOC726007, LOC551754, LOC412192, LOC550698, LOC100576975, LOC727634, LOC412069, LOC552284, LOC412398, LOC100578892, LOC725039, LOC411989, LOC102656541, LOC551376, LOC100578450, LOC107964839, LOC724870, LOC102656792, LOC409637, LOC726778, LOC551888, LOC412504, LOC726811, LOC726822, LOC409353,* ***Hex70a****,* ***HEX70b*** |
| 9 | 159,406 | 823,876 | 664.47 | 0 | 12 | *LOC727173, LOC409268, LOC409388, LOC102655897, LOC409433, LOC412318, LOC102655064, LOC107964022, LOC102656465, LOC107965817, LOC102655120, LOC100576187* |
| 9 | 842,007 | 1,263,372 | 421.37 | 2 | 21 | *LOC100576187, LOC727568, LOC551305, LOC100576085, LOC102656577, LOC409281, LOC725598, LOC551373, LOC113218991, LOC107965827, LOC726444,* ***Grp,*** *LOC107965793, LOC725842, LOC113219003, LOC107965829, LOC113219005, LOC113218983, LOC725031, LOC412679, LOC412665, LOC102654952,* ***Rep*** |
| 9 | 1,459,383 | 1,811,708 | 352.33 | 0 | 41 | *Mir6006, LOC107965007, LOC412647, LOC727153, LOC727157, LOC726740, LOC552467, LOC409741, LOC552273, LOC102654498, LOC552291, LOC551554, LOC551576, LOC551907, LOC107965832, LOC551936, LOC100576439, LOC102656685, LOC412510, LOC100576436, LOC411960, LOC550780, LOC551445, LOC725807, LOC102656492, LOC550692, LOC551097, LOC100576348, LOC727599, LOC411653, LOC409144, LOC725563, LOC413984, LOC113218977, LOC100578763, LOC725558, LOC551433, LOC412446, LOC102656849, LOC412787, LOC113218985* |
| 9 | 1,812,584 | 1,983,997 | 171.41 | 1 | 8 | *LOC412787, LOC102656780, LOC726239,* ***WRNexo****, LOC413692, LOC550940, LOC727278, LOC412688, LOC726367* |
| 11 | 4,116,645 | 4,546,201 | 429.56 | 0 | 22 | *LOC552774, LOC727303, LOC552779, LOC413772, LOC726443, LOC412663, LOC411585, LOC725069, LOC552002, LOC411811, LOC727122, LOC727290, LOC412423, LOC725316, LOC113219102, LOC100578943, LOC409438, LOC727299, LOC107965795, LOC113219105, LOC727510, LOC726993* |
| 11 | 4,562,764 | 4,701,360 | 138.60 | 0 | 3 | *LOC726993, LOC725680, LOC100577161* |
| 11 | 4,752,441 | 4,828,081 | 75.64 | 0 | 3 | *LOC113219081, LOC113219082, LOC102654883* |
| 11 | 4,828,511 | 4,959,139 | 130.63 | 0 | 2 | *LOC113219114, LOC113219087* |
| 11 | 4,977,728 | 5,265,258 | 287.53 | 0 | 8 | *LOC100578194, LOC552407, LOC411535, LOC102656914, LOC552348, LOC413728, LOC726770, LOC100578332* |
| 11 | 6,981,691 | 7,146,872 | 165.18 | 0 | 24 | *LOC100578112, LOC551483, LOC412278, LOC550881, LOC100578723, LOC107965805, LOC413657, LOC724622, LOC409178, LOC724527, LOC411718, LOC551148, LOC411719, LOC724446, LOC552480, LOC102655030, LOC107965271, LOC413827, LOC552085, LOC552056, LOC412606, LOC551379, LOC102655272, LOC409470* |
| 11 | 14,823,658 | 15,003,563 | 0.180 | 0 | 37 | *LOC551424, LOC724842, LOC724877, LOC409595, LOC409877, LOC552769, LOC100577344, LOC412219, LOC727234, LOC409393, LOC552744, LOC727222, LOC551313, LOC552733, LOC411505, LOC409491, LOC552714, LOC413822, LOC552700, LOC551276, LOC410108, LOC727221, LOC412046, LOC409329, LOC100576647, LOC551964, LOC107965281, LOC725781, LOC409241, LOC411861, LOC411862, LOC411863, LOC409636, LOC409634, LOC102653856, LOC100576717, LOC551467* |
| 15 | 2,635,510 | 2,869,345 | 233.84 | 1 | 33 | *LOC410040, LOC409137, LOC552795, LOC725201, LOC409513, LOC100577640, LOC552784, LOC552479, LOC727622, LOC100577797, LOC551413, LOC411897, LOC552028, LOC552053, LOC552082, LOC411742, LOC552155, LOC100578262, LOC409447, LOC551194, LOC551237, LOC550659, LOC725379, LOC100577909,* ***Snf****, LOC102656197, LOC100578560, LOC413517, LOC725394, LOC413515, LOC113219277, LOC725503, LOC552258, LOC725584* |
