# Supplementary material for: Identification of runs of homozygosity in Western honey bees (Apis mellifera) using whole‐genome sequencing data
Source: Ecol Evol. 2023 Jan 17;13(1):e9723. doi: 10.1002/ece3.9723 (PMC9843643; doi:10.1002/ece3.9723)
Supplement: Supplementary file 4 — Table S3 [file ECE3-13-e9723-s001.docx]

Table S3 : List of annotated and uncharacterised loci within homozygosity islands of Apis mellifera carnica with admixture proportions < 10% (n=12)

| **Chr.** | **Begin (bp)** | **End (bp)** | **Length (Kb)** | **N annot. genes** | **N. unchar. loci** | **Characterised genes and uncharacterised loci** |
| --- | --- | --- | --- | --- | --- | --- |
| 11 | 4235653 | 4289234 | 53.58 | 0 | 6 | LOC552774, LOC727303, LOC552779, LOC413772, LOC726443, LOC412663 |
| 11 | 4289611 | 4788504 | 498.89 | 0 | 21 | LOC412663, LOC411585, LOC725069, LOC552002, LOC411811, LOC727122, LOC727290, LOC412423, LOC725316, LOC113219102, LOC100578943, LOC409438, LOC727299, LOC107965795, LOC113219105, LOC727510, LOC726993, LOC725680, LOC100577161, LOC113219081, LOC113219082 |
| 11 | 4789329 | 4827021 | 37.69 | 0 | 2 | LOC113219082, LOC102654883 |
| 11 | 4827654 | 5174776 | 347.12 | 0 | 10 | LOC113219114, LOC113219087, LOC100578194, LOC552407, LOC411535, LOC102656914, LOC552348, LOC413728, LOC726770, LOC100578332 |
| 11 | 5230911 | 5287003 | 56.09 | 0 | 1 | LOC100578332 |
| 11 | 5804079 | 5837418 | 33.34 | 0 | 1 | LOC724287 |
| 11 | 5871758 | 6016851 | 145.09 | 0 | 1 | LOC724287 |
| 11 | 6083442 | 6108833 | 25.39 | 0 | 1 | LOC724287 |
| 11 | 6109355 | 6294348 | 184.99 | 0 | 6 | LOC724287, LOC113219127, LOC550987, LOC107965233, LOC551883, LOC102653967 |
| 11 | 6294671 | 6757043 | 462.37 | 5 | 31 | LOC551883, LOC102653967, LOC409622, LOC107965288, LOC102655440, LOC113219115, LOC102655983, LOC107965752, LOC409784, LOC409841, LOC100577578, LOC102655203, LOC100578061, LOC100578030, LOC100577766, LOC727192, **CPR1, CPR2, CPR3, CPR4,** LOC107965751, LOC410134, LOC724715, LOC724670, LOC102654871, LOC552814, LOC102654700, **Rga**, LOC552823, LOC551635, LOC551668, LOC724942, LOC550884, LOC550926, LOC409494, LOC727170 |
| 11 | 6797742 | 7082258 | 284.52 | 0 | 25 | LOC412536, LOC552680, LOC100577649, LOC412346, LOC412222, LOC551140, LOC552154, LOC727205, LOC100579022, LOC100578112, LOC551483, LOC412278, LOC550881, LOC100578723, LOC107965805, LOC413657, LOC724622, LOC409178, LOC724527, LOC411718, LOC551148, LOC411719, LOC724446, LOC552480, LOC102655030 |
